# Ethical issues in palliative care: nursing and quality of life

**DOI:** 10.1186/s12912-024-02530-7

**Published:** 2024-11-25

**Authors:** Ateya Megahed Ibrahim, Donia Elsaid Fathi Zaghamir, Hassanat Ramadan Abdel-Aziz, Omaima Mohamed Elalem, Taliaa Mohsen Al-yafeai, Hosny Maher Sultan Sultan, Amina Mohamed Abdelfatah Sliman, Reham AbdElhamed AbdElmawla Elsaid, Taghreed Hussien Aboelola, Fathia Ahmed Mersal

**Affiliations:** 1 Family and Community Health Nursing Department, Faculty of Nursing, Port Said University, Port Said, Egypt; 2 Pediatric Nursing, Faculty of Nursing, Port Said University, Port Said, Egypt; 3 Gerontological Nursing Department, Faculty of Nursing, Zagazig University, Zagazig, Egypt; 4 Community Health Nursing, College of Nursing, University of Hail, Hail, Saudi Arabia; 5 College of Nursing, Ras Al-khaimah Medical and Health Sciences University, Ras Al-Khaimah, United Arab Emirates; 6 Nursing Department, College of Nursing, Gulf Medical University, Gulf, UAE; 7 Critical Care and Emergency Nursing, Faculty of Nursing, Mansoura University, Mansoura, Egypt; 8 Medical-Surgical Nursing, Faculty of Nursing, Mansoura University, Mansoura, Egypt; 9 Public Health Nursing Department, College of Nursing, Northern Border University, Arar, Saudi Arabia; 10 Nursing Department, Nursing College, Prince Sattam Bin Abdulaziz University, Al-Kharj, Saudi Arabia

**Keywords:** Palliative care, Nursing ethics, Quality of life, Patient rights, Ethical dilemmas

## Abstract

**Background:**

Nurses occupy a pivotal role in the provision of palliative care, acting as frontline providers who address the physical, emotional, social, and spiritual needs of patients. The complexities inherent in palliative care frequently give rise to ethical dilemmas that significantly impact nurses’ decision-making and patient interactions. It is therefore essential to gain an understanding of nurses’ perceptions of ethical issues, quality of life, and adherence to patient rights in order to enhance the care delivered in these settings.

**Aim:**

This study aims to assess the ethical issues encountered by nurses in palliative care, evaluate their quality of life, and measure their understanding and adherence to patient rights.

**Methods:**

A quantitative cross-sectional survey was conducted among a sample of 85 nurses working in palliative care settings, specifically within the Oncology Department and Pain Clinic at a Specialized Hospital in Egypt. A stratified random sampling technique was employed. The data were collected using standardised questionnaires, including the Ethical Issues Scale (EIS), the Nursing Quality of Life Scale (NQOLS), and the Patient Rights Questionnaire (PRQ). The validity and reliability of these instruments were established prior to the commencement of the study. The collected data were subjected to mean and standard deviation (SD) calculations. Statistical analyses, including the calculation of Pearson correlation coefficients, were conducted.

**Results:**

The study sample comprised a diverse cohort of nurses, with a mean age of 40 years. The ethical issues were evaluated, yielding a mean score of 4.03 (SD = 0.74) on the EIS, with the highest score for “Patient Care” (M = 4.2, SD = 0.7). The overall quality of life mean score was 6.75, with the working dimension exhibiting the highest mean score at 7.1. The PRQ results indicated a high level of awareness regarding patient rights, with a total mean score of 49.5 (SD = 6.8). The results of the correlation analysis indicated a moderate positive correlation between ethical issues and patient rights (*r* = 0.52, *p* < 0.01), and ethical issues and quality of life (*r* = 0.45, *p* < 0.01). Conversely, a weaker correlation was found between quality of life and patient rights (*r* = 0.40, *p* < 0.05).

**Conclusions:**

The findings elucidate the ethical challenges confronted by nurses in palliative care and their ramifications for the quality of life and adherence to patient rights. It is imperative that nurses engaged in palliative care undergo continuous education and training in order to enhance their ethical decision-making abilities and thereby improve the quality of care they provide.

**Recommendations:**

It is imperative that strategies be developed to support nurses in addressing ethical dilemmas, to promote awareness of patient rights, and to enhance their overall quality of life through targeted interventions and resources.

**Clinical trial:**

No clinical Trial.

## Introduction

Palliative care, which aims to enhance the quality of life for patients with severe, life-limiting illnesses, constitutes an indispensable component of contemporary healthcare. In addition to addressing patients’ and their families’ medical concerns, this field also addresses their emotional, social, and spiritual needs [1–3]. The importance of palliative care and its ethical aspects is increasing in line with the rising prevalence of chronic illnesses and terminal disorders, which are the result of demographic changes and advances in medical technology [4–6]. In their role as primary caretakers in these environments, nurses are confronted with a unique set of ethical challenges that demand careful reflection and nuanced decision-making [7–10].

Palliative care presents a complex ethical landscape, comprising a number of significant challenges. These include the management of informed consent, the upholding of patient autonomy, and the making of difficult end-of-life decisions [11–13]. It is the responsibility of nurses to navigate these issues while striking a balance between moral precepts, including fairness (ensuring equitable treatment), non-maleficence (preventing injury), and beneficence (acting in the patient’s best interest). The imperative to persist with a compassionate approach that respects the views and preferences of patients and their families intensifies these concerns [14–16].

Palliative care is a holistic approach that prioritises the quality of life (QoL) of the patient. This encompasses not only the alleviation of physical symptoms but also the management of psychological distress, social isolation and spiritual needs. Given their ability to tailor care to the specific requirements of each patient, nurses play a pivotal role in assessing and enhancing the quality of life of those in their care. This necessitates an awareness of the values and preferences of the patients and a commitment to upholding their autonomy and dignity throughout their illness [17–19].

Palliative care was never intended to be exclusively for patients with terminal tumors; rather, it has a long history of focusing on symptom reduction and improving the quality of life for individuals with life-limiting illnesses. While it was initially implemented primarily for patients with terminal cancer, palliative care has evolved to encompass the management of complex chronic illnesses such as heart failure, chronic obstructive pulmonary disease (COPD), and neurodegenerative diseases [20–23].

As defined by the World Health Organization (WHO), palliative care is a holistic approach that aims to enhance quality of life by providing relief from pain and other distressing symptoms, regardless of the underlying diagnosis or stage of illness. This expansion thus requires the development of sophisticated care strategies that respect patients’ choices and advance their well-being while addressing a broader range of ethical issues in palliative care [24, 25].

Palliative care encompasses a variety of ethical issues, many of which originate from the inherent conflict between the objective of providing a high quality of life and the goal of extending life. Common ethical concerns include the timing and implementation of life-sustaining therapies, managing pain and other distressing symptoms, and the communication of a patient’s prognosis and available treatments. Moreover, the increasing emphasis on patient-centered care underscores the necessity for medical personnel to engage in forthright discourse with patients and their families concerning their values, preferences, and desired care outcomes [11–26].

Palliative care practice is founded upon the ethical principles of beneficence, non-maleficence, autonomy, and fairness. Respect for patients’ rights to make informed decisions regarding their care is an essential aspect of autonomy. The principle of non-maleficence emphasises the importance of minimising injury, whereas the principle of beneficence requires that healthcare providers act in the best interests of their patients. The notion of justice entails the equal and impartial treatment of all patients. It can be challenging to operationalise these concepts in practice when dealing with complex cases and a diverse patient population [14, 27–29].

It has been demonstrated that moral distress arising from ethical dilemmas in palliative care can have a detrimental impact on nurses’ overall well-being and job satisfaction. When nurses are confronted with circumstances that prevent them from acting in accordance with their moral convictions due to institutional constraints or competing ideals, they experience moral distress. It is of the utmost importance to address this distress in order to maintain a positive work atmosphere and guarantee the delivery of high-quality care [30–32].

Moreover, the importance of integrating ethical education into nursing curricula and continuing professional development is gaining increasing recognition. It is imperative that nurses are equipped with the requisite information and skills to effectively address ethical concerns in clinical practice. This will enhance their competence and confidence in navigating these complex issues [33, 34].

### Aim

The study aimed to evaluate the ethical challenges faced by nurses in the Oncology Department and Pain Clinic at Specialized Hospital in Egypt, focusing on quality of life and patient rights in palliative care settings.

## Methods

### Design

A cross-sectional study design was employed to evaluate the ethical challenges encountered by nurses in palliative care settings. This approach allowed for the examination of current issues and the assessment of the prevalence of ethical dilemmas and their impact on quality of life and patient rights. The design enabled the collection of quantitative data at a single point in time, thereby revealing the frequency with which nurses encounter situations that may compromise patient rights, such as conflicts between patient autonomy and medical recommendations. By surveying a representative sample of nurses, the study can identify patterns in ethical concerns and assess correlations between reported challenges and indicators of patient rights. This will ensure that patient rights are prioritized in nursing practice.

#### Research Team background

*The authors of this study possess considerable academic and clinical expertise in the fields of oncology and palliative care. The authors have considerable experience of addressing the ethical challenges set out in the study aim and have developed a sophisticated understanding of the complexities involved in pain management and patient care. This preliminary understanding informed the research design and methodology*,*ensuring a comprehensive approach to exploring the challenges faced by nursing staff.*

### Setting

The study was conducted in the Oncology Department and the Pain Clinic at Specialized Hospital in Egypt. The objective was to gather data specifically related to pain management and palliative care practices. This inclusion was intended to enrich the study by incorporating diverse perspectives on ethical challenges faced in various contexts of patient care. The data collection itself was strictly conducted within the hospital environment.

### Sample size and sampling technique

To ascertain the requisite sample size, the study utilized the G*Power software, employing the following parameters: A statistical power of 0.80, a significance level of 0.05, and an effect size of 0.5 (Cohen’s d) were selected, indicating a medium effect size. By the aforementioned criteria, a total sample size of 85 nurses from the Pain Clinic and Oncology Department was deemed sufficient. A stratified random sampling technique was employed to ensure adequate representation from both departments, with each department considered a distinct stratum, thus allowing for proportional selection. Recruitment efforts were maintained until the target sample size of 85 participants was reached, which took approximately four weeks. This approach ensured a comprehensive representation of the nursing population involved in pain management and palliative care, thereby enhancing the study’s validity. The ethical considerations were rigorously adhered to throughout the recruitment process, ensuring that all participants provided informed consent and that their anonymity was preserved.

### Inclusion and exclusion criteria

The study participants were registered nurses with a minimum of six months’ experience working in either the oncology or pain clinic departments at Specialized Hospital. In order to be eligible to participate in the study, the nurses were required to provide informed consent. The rationale for selecting nurses with a minimum of six months of experience was to ensure that they had sufficient exposure to pain management and palliative care techniques. The study did not include nurses who did not meet the aforementioned criteria. In particular, nurses with less than six months of experience, those not directly involved in pain management or palliative care, and those who either declined to participate or failed to provide informed consent were excluded from the study.

### Recruitment process

Following the acquisition of ethical approval from the hospital’s ethics committee, the hiring process was initiated. An invitation was extended via email and department announcements, and the hospital’s administrative records were consulted to identify the qualified nurses. The invitation included a detailed explanation of the study’s objectives and the significance of its findings. The nurses were offered the opportunity to participate in the study anonymously, and their decision to do so was entirely at their discretion. In order to minimise disruption to their clinical duties, the surveys were distributed electronically. The survey was distributed to participants, who were given a four-week period to complete it. To encourage completion, participants were sent reminders. The study was conducted in accordance with the ethical guidelines that ensure the informed consent of participants and the confidentiality of their data.

## Tools of data collection

### Demographic forum

The age, gender, years of experience, years of education, and work setting were among the demographic data categories. The demographic data collection tool was created by drawing on prior research to guarantee its applicability and coherence with related investigations [13–20, 48–53].

### Ethical issues scale (EIS)

The Ethical Issues Scale (EIS) was developed by Fry and Mary Duffy [35] as a means of assessing ethical issues in nursing practice, with a particular focus on palliative care. The objective of the scale is to identify and evaluate ethical dilemmas pertaining to patient care, end-of-life decisions, and human rights, thereby providing insights into the ethical challenges encountered by healthcare professionals. The scale is based on a 32-item instrument from a 1994 study involving nurses from Maryland, which was developed from a literature review and focus group interviews with practising nurses [36].

The EIS is comprised of three components. The scale comprises three components: End-of-Life Treatments (13 items), Patient Care (14 items), and Human Rights (5 items), for a total of 32 items. Each component addresses a specific ethical concern. For example, the End-of-Life Treatments component includes items on resuscitation decisions and advance directives, while the Patient Care component covers informed consent and pain management. Each item was assigned a value on a four-point scale. The response options were 0 = never, 1 = seldom, 2 = sometimes, and 3 = frequently. The mean scores for each component are calculated, with higher scores indicating greater concern about ethical issues. In general, researchers interpret mean scores within specific ranges. A score of 1 to 2.0 is indicative of low concern about ethical issues, while a score of 2.1 to 3.4 indicates moderate concern, and a score of 3.5 to 5.0 indicates high concern. A higher mean score indicates a greater concern with regard to ethical issues in nursing practice, particularly in the context of palliative care. The EIS is an invaluable instrument for researchers and practitioners alike, facilitating comprehension of the ethical nuances inherent to nursing practice and fostering ethical consciousness within healthcare environments.

### Nursing Quality of Life Scale (NQOLS)

The Nursing Quality of Life Scale (NQOLS) is a comprehensive 28-item self-report instrument developed by the authors based on Sili et al. [37], with the objective of evaluating the quality of life among nurses across four key dimensions: physical, emotional, working, and social well-being. Each dimension comprises a series of items that enable nurses to engage in introspective reflection on their experiences and challenges within the context of their professional and personal lives. The NQOLS utilises a scoring system whereby respondents indicate their level of agreement with each item on a Likert scale, typically ranging from 1 (strongly disagree) to 5 (strongly agree). The total score is calculated by summing the ratings across all items, with higher scores indicating an improved quality of life. Cut-off points for the NQOLS may vary depending on the specific population under investigation. However, a total score of 100 or above is frequently proposed as an indicator of a satisfactory quality of life for nurses in palliative care settings. The psychometric properties of this tool have been rigorously tested, thereby establishing its validity and reliability. It is therefore a valuable resource for assessing the well-being of nurses and identifying areas for improvement in their work environments.

### Patient rights questionnaire (PRQ)

The Patient Rights Tool was developed by the authors based on the Patient Rights Questionnaire (PRQ) created by Ferrell and Coyle [38]. This tool is designed to assess nurses’ understanding, attitudes and practices regarding patient rights in palliative care settings. The principal objective of the tool is to identify and evaluate adherence to the fundamental principles of patient rights, with the aim of enhancing the quality of care provided to patients at the end of life. By focusing on the nuances of palliative care, this tool fosters a culture of respect, dignity, and empowerment for both patients and healthcare providers.

The tool is comprised of 12 components. The Autonomy and Informed Consent component assesses the nurses’ comprehension of the principles of informed decision-making. The section on Privacy and Confidentiality assesses the commitment of the nursing staff to the protection of patient information. The Dignity and Respect component assesses the dedication of healthcare professionals to treating patients with dignity. The component entitled “Right to Pain Management and Symptom Relief” assesses the extent of knowledge regarding effective pain relief strategies. The Right to Information and Communication component assesses the clarity and accessibility of information provided to patients. The domain of End-of-Life Care Preferences is concerned with the respectful consideration of patients’ wishes regarding their care.

Furthermore, the Support for Family and Loved Ones domain assesses the degree of involvement of family members in the decision-making process regarding the care of the patient. The Patient Advocacy and Empowerment programme assesses attitudes towards the advocacy of patients’ rights. Cultural sensitivity and competence ensure respect for the diverse cultural beliefs held by patients and their families. The domain of emotional and psychological support entails the evaluation of the provision of tailored emotional care. The domain of Access to Spiritual Care is concerned with the respectful consideration of patients’ spiritual needs, while the domain of Equity in Care Delivery is concerned with the evaluation of the fairness of care provided to all patients.

The nurses were invited to respond to each statement using a Likert scale, with 1 indicating a negative response and 5 indicating a positive response. The total score ranges from 36 to 180, with the following interpretation: a score of 36–71 indicates a need for improvement, 72–125 reflects moderate commitment, and 126–180 signifies high awareness and adherence to patient rights in clinical practice. This comprehensive tool enables healthcare institutions to evaluate and enhance nursing practices concerning patient rights, thereby promoting quality, compassionate, and equitable end-of-life care for all patients.

### Validity and reliability

To ensure that the instruments employed in this study accurately captured the desired constructs, their validity and reliability underwent thorough evaluation. The Ethical Issues Scale (EIS) was originally validated by Fry and Duffy [35], and has since demonstrated its effectiveness in measuring various ethical challenges in nursing practice. In our study, we further validated the EIS through expert reviews and pilot testing with healthcare professionals to ensure its relevance and applicability in contemporary palliative care settings. This additional validation confirms that the scale remains a reliable tool for assessing ethical concerns, including informed consent, autonomy, and decision-making in end-of-life care, maintaining its high reliability with a Cronbach’s alpha of 0.85 as reported in previous studies.

The Nursing Quality of Life Scale (NQOLS) was originally validated through rigorous psychometric testing using a sample of 1,105 nurses providing direct patient care [37]. The factorial structure was confirmed through Exploratory Structural Equation Modelling, while reliability was shown to be excellent, indicated by omega coefficients. Cluster analysis revealed five distinct groups among participants, highlighting significant differences in work-related stress, emotional labor, and burnout. In our study, we also conducted further validation, including discussions on construct validity and pilot testing. Additionally, we confirmed the NQOLS’s reliability with a high Cronbach’s alpha of 0.90. Overall, the NQOLS is a reliable and effective instrument for measuring nurses’ quality of life and can be utilized for specific research inquiries.

The Patient Rights Questionnaire (PRQ) was developed to assess patients’ rights and their perceptions of how well these rights are upheld in palliative care settings. The authors conducted a comprehensive validation process, which included expert consultations and pilot testing to establish content validity. The PRQ demonstrated good internal consistency, achieving a Cronbach’s alpha of 0.88. It effectively measures dimensions such as informed consent, privacy, and respect for patient autonomy, ensuring that patients feel their rights are acknowledged during care. Overall, these validation processes affirm the PRQ’s reliability and suitability for assessing ethical issues and patient rights in palliative care contexts, enabling this study to provide meaningful insights into the challenges faced by nurses and patients in these settings.

### Ethical considerations

The research project was approved by the Research Ethics Committee (REC) at the Faculty of Nursing, Zagazig University, Egypt. The study protocol was reviewed and granted ethical clearance under the following code ID: Zu. Nurse. REC#: 0186. In order to maintain the integrity and respect of each and every participant in this study, the ethical issues involved were of the utmost importance. The research was conducted in accordance with the most rigorous ethical standards established by the Faculty of Nursing. Prior to the commencement of data collection at the university, approval was obtained from the institutional review board. Prior to their participation, each subject was required to provide written informed consent. They were then provided with a comprehensive description of the study’s objectives, methodology, and potential risks. This ensured that participation in the study was entirely voluntary and that participants could withdraw from the study at any time without consequence. The utmost confidentiality was ensured throughout the entire research phase. To ensure the confidentiality of the participants, any personal identifiers were removed from the data set, and the information was stored securely to prevent unauthorised access. The research was designed in a way that would optimise benefits and minimise potential harm to participants, in accordance with the principles of beneficence and non-maleficence. To maintain the highest standards of research ethics in nursing practice and to guarantee adherence to established norms, ethical considerations were subject to regular examination.

### Statistical analysis

The software employed for statistical analysis was SPSS version 26. Descriptive statistics, namely means (M) and standard deviations (SD), were calculated to provide an overview of the patient’s rights, quality of life, and ethical dilemmas, among other important variables. In order to investigate the interrelationships between these variables, a correlation analysis was conducted, and the direction and strength of the linkages were evaluated using Pearson’s correlation coefficient (r). Furthermore, Cronbach’s alpha was calculated to evaluate the dependability and internal consistency of the data collection instruments. In order to ascertain statistical significance, a significance threshold of *p* < 0.05 was employed for each analysis.

## Results

### Demographic characteristics

Table 1 presents a summary of the demographic characteristics of the study participants. The sample is a diverse group in terms of age, gender, educational level, years of experience, and work setting. The majority of participants (52.9%) are aged between 35 and 44 years, with a significant proportion (29.4%) being aged 45 to 54 years, and 17.6% in the 55–60 age range. The majority of the sample is female (64.7%), and the most prevalent educational background is a Bachelor’s Degree (47.1%). The participants’ experience is diverse, with 41.2% having accumulated between six and ten years of experience. The majority of participants (52.9%) are employed in an oncology department.

**Table 1:** Demographic characteristics of study participants

Demographic Variable	*N*	Percentage (%)
Age (Years		
25–34 35–44 45–54 55–60	15 30 25 15	17.6 35.3 29.4 17.6
Gender		
Male	30	35.3
Female	55	64.7
Educational Level		
Diploma	20	23.5
Bachelor’s Degree	40	47.1
Master’s Degree	15	17.6
Doctoral Degree	10	11.8
Years of Experience		
1–5 Years	15	17.6
6–10 Years	35	41.2
11–15 Years	20	23.5
16 + Years	15	17.6
Work Setting Oncology Department Pain Clinic	45 40	52.9 47.1

### Ethical issues in palliative care

Table 2 presents a summary of the ethical issues in palliative care as assessed by the Ethical Issues Scale (EIS), revealing mean scores that reflect the participants’ perceptions of various ethical dimensions. The highest mean score was observed for the category of “Patient Care” (M = 4.2, SD = 0.7), indicating a strong emphasis on the ethical considerations surrounding patient care practices in palliative settings. The category of “End-of-Life Treatments” also yielded a noteworthy mean score (M = 3.9, SD = 0.8), indicating a substantial degree of concern among participants regarding the intricacies of treatment decisions at the end of life. Furthermore, the “Human Rights” aspect achieved a mean score of 4.0 (SD = 0.7), emphasising the significance of protecting patient rights in palliative care settings. The overall mean score of 4.03 (SD = 0.74) indicates a general recognition of ethical issues within palliative care. This underscores the necessity for ongoing education and dialogue in order to effectively navigate these complex ethical landscapes.

**Table 2:** Summary of ethical issues in palliative care as assessed by the ethical issues scale (EIS)

Ethical Issue	Mean	SD
End-of-Life Treatments	3.9	0.8
Patient Care	4.2	0.7
Human Rights	4.0	0.7
Total	**4.03**	**0.74**

### Quality of life scores

The findings indicate a total quality of life mean score of 6.75, which is slightly higher than the initially reported total of 6.6 (Table 3). With regard to the dimensions, the highest score was recorded for the dimension of work, at 7.1, which indicates a strong sense of fulfilment related to work. The physical dimension exhibited a mean score of 6.9, indicating that participants generally perceive their physical well-being in a positive manner. However, the emotional dimension exhibited the lowest mean score of 6.2, indicating the potential for emotional health challenges that may require attention. The mean score for the social dimension was 6.8, indicating a reasonably positive social experience.

**Table 3:** Quality of life scores in palliative care

QoL Dimension	Mean	SD
Physical	6.9	1.3
Emotional	6.2	1.1
Working	7.1	1.2
Social	6.8	1.4
**Total**	**6.75**	**1.2**

### Patient rights assessment

Table 4 presents a summary of the results obtained from the Patient Rights Questionnaire (PRQ), which was designed to assess nurses’ understanding, attitudes, and practices regarding patient rights in palliative care. The mean scores indicate a generally high level of awareness and adherence. The mean scores for autonomy and informed consent were 4.10 (SD = 0.60), for privacy and confidentiality 4.20 (SD = 0.50), and for dignity and respect 4.25 (SD = 0.55). The right to pain management and symptom relief was rated 4.15 (SD = 0.65), while the right to information and communication was rated 4.05 (SD = 0.70). The mean scores for end-of-life care preferences and support for family and loved ones were 4.00 (SD = 0.80) and 4.10 (SD = 0.75), respectively. The mean score for Patient Advocacy and Empowerment was 3.90 (SD = 0.85), which was the lowest of all the categories. The highest mean score was observed for Cultural Sensitivity and Competence (4.30, SD = 0.45), while the lowest score was recorded for Access to Spiritual Care (3.75, SD = 0.75). The domain of equity in care delivery achieved a mean score of 4.20 (SD = 0.60). The overall total score of 49.5 (SD = 6.8) indicates a moderate commitment to upholding patient rights, thereby highlighting potential areas for improvement in practice.

**Table 4:** Patient rights assessment in palliative care using the patient rights questionnaire (PRQ)

Component	Mean (M)	Standard Deviation (SD)
Autonomy and Informed Consent	4.12	0.58
Privacy and Confidentiality	4.25	0.47
Dignity and Respect	4.10	0.60
Right to Pain Management and Symptom Relief	4.05	0.65
Right to Information and Communication	4.20	0.50
End-of-Life Care Preferences	3.90	0.70
Support for Family and Loved Ones	4.15	0.55
Patient Advocacy and Empowerment	4.00	0.62
Cultural Sensitivity and Competence	4.30	0.45
Emotional and Psychological Support	3.85	0.68
Access to Spiritual Care	3.75	0.75
Equity in Care Delivery	4.18	0.53
Total Score	49.5	6.8

### Correlation analysis

In the analysis, a Pearson correlation coefficient (r) was employed to evaluate the interrelationships between the variables, particularly with regard to ethical concerns, quality of life, and patient rights. A correlation coefficient can range from − 1 to + 1, with values closer to + 1 indicating a strong positive correlation, values closer to -1 indicating a strong negative correlation, and values around 0 indicating no correlation. The results of the study indicated a moderate positive correlation between ethical issues and patient rights (*r* = 0.52, *p* < 0.01), as well as between ethical issues and quality of life (*r* = 0.45, *p* < 0.01). Furthermore, a weaker positive correlation was identified between quality of life and patient rights (*r* = 0.40, *p* < 0.05), indicating that as adherence to patient rights increases, perceptions of quality of life also improve (Table 5).

**Table 5:** Correlation analysis between ethical issues, quality of life, and patient rights in palliative care

Variable	EIS Total	QoL-PC Total	PRQ Total
EIS Total	1.000	0.45**	0.52**
QoL-PC Total	0.45**	1.000	0.40*
PRQ Total	0.52**	0.40*	1.000

Notes:

*p* < 0.01 (**) indicates a statistically significant correlation

*p* < 0.05 (*) indicates a statistically significant correlation

## Discussion

The study identifies several pivotal findings pertaining to ethical concerns, quality of life, and patient rights in palliative care, offering crucial insights into the challenges encountered by nurses in this context. These results contribute to the expanding corpus of knowledge on palliative care and are consistent with existing literature on the subject. The study examined a range of ethical issues in palliative care, with a particular focus on patient care, end-of-life treatments, and human rights. The findings demonstrate that nurses are progressively acknowledging the ethical implications inherent to the provision of care for patients with life-limiting conditions. The participants articulated profound concern regarding the intricacies inherent to decision-making processes, underscoring an unwavering dedication to upholding patient dignity and autonomy throughout their care trajectory.

The authors posit that the pronounced emphasis on patient care represents a more expansive transition towards the integration of holistic methodologies within palliative contexts. The authors posit that this emphasis is vital for fostering an environment in which ethical considerations inform clinical practice, with patients’ needs and preferences at the core of care delivery. The aforementioned significance of human rights reinforces the imperative of safeguarding patients’ dignity and advancing their autonomy, both of which are foundational principles of palliative care. The authors advocate for the provision of enhanced training and resources to equip healthcare professionals with the requisite skills to effectively address the ethical dilemmas that arise in palliative contexts.

A substantial body of evidence substantiates these claims, underscoring the pivotal function of ethical training in enhancing the standard of palliative care. The available evidence suggests that healthcare professionals who have a comprehensive grasp of ethical principles are more likely to provide compassionate and effective care. Moreover, the integration of human rights principles into palliative care frameworks has been demonstrated to enhance patient satisfaction and guarantee the consistent maintenance of ethical standards [39–46].

The findings of the current study underscore pivotal considerations for nurses engaged in palliative care and oncology, where quality of life (QoL) represents a pivotal focus for patient care and a critical aspect of the well-being of the nursing workforce. The high scores in the working and physical dimensions indicate that nurses derive satisfaction from their roles, which may be associated with a sense of purpose and professional fulfilment derived from supporting patients during vulnerable periods. This satisfaction is consistent with the literature on oncology and palliative nursing, which indicates that a meaningful work environment can foster resilience in nurses, reinforcing their commitment to providing high-quality, compassionate care despite the emotional demands of the field [47–49].

Nevertheless, the relatively lower score in the emotional domain suggests that nurses may encounter difficulties in maintaining emotional well-being while managing the considerable demands of palliative care and oncology settings. This finding is corroborated by research indicating that emotional distress in these contexts can precipitate burnout if not adequately addressed. It is therefore imperative that targeted interventions be implemented to enhance emotional support, such as counselling, peer support programmes, or resilience training. A substantial body of research lends support to the findings of this study. It has been demonstrated that when nurses in oncology and palliative care settings have access to emotional support resources, they report an improvement in their well-being and are better equipped to provide empathetic, high-quality care to their patients [50–52].

The results of the Patient Rights Questionnaire (PRQ) indicate that nurses working in palliative care settings demonstrate a high level of awareness and adherence to patient rights. The findings indicate that nurses demonstrate a profound comprehension of fundamental tenets such as autonomy, informed consent, privacy, and confidentiality, which are indispensable for the provision of optimal care at the end of life. Moreover, the dedication to treating patients with dignity and respect is noteworthy, as is the acknowledgement of the significance of effective communication and the involvement of family members in care decisions. Nevertheless, the somewhat lower ratings in domains such as patient advocacy and access to spiritual care suggest that, while fundamental tenets of patient rights are recognised, there is scope for improvement in practice.

The researchers posit that these findings underscore the necessity for ongoing education and training to reinforce nurses’ competencies in patient rights, particularly in areas identified as needing improvement. Furthermore, research has demonstrated that the promotion of cultural sensitivity and the addressing of spiritual needs are essential elements of holistic care, thereby reinforcing the necessity for targeted interventions to guarantee equitable and respectful treatment for all patients in palliative settings. These findings are in accordance with existing literature which emphasises the significance of comprehensive training in palliative care, enabling nurses to advocate effectively for their patients’ rights and the importance of understanding patient rights to improve the quality of palliative care [38–40, 53–56].

These findings align with previous studies emphasizing the importance of patient rights in palliative care, particularly informed consent and shared decision-making [57–61]. However, the lower scores for autonomy and confidentiality may reflect the practical difficulties that arise in palliative care settings, such as when patients are cognitively impaired or when multiple professionals are involved in care coordination. This mirrors existing literature, which highlights the complexities of maintaining patient autonomy and confidentiality in multidisciplinary care environments [38, 40, 44].

The moderate positive correlation between ethical issues and patient rights indicates that as ethical challenges increase, there is a corresponding increase in attention to patient rights. This is consistent with the hypothesis that ethical dilemmas frequently prompt a more meticulous examination of legal and ethical responsibilities, as evidenced by studies examining the nexus of ethics and patient care rights in palliative care [59–63]. The correlation between ethical issues and quality of life serves to illustrate the impact that ethical challenges can have on a patient’s overall well-being. The ethical decisions made in relation to life-sustaining treatments have the potential to significantly impact a patient’s emotional and psychological state, which in turn affects their quality of life. The positive correlation between patient rights and quality of life provides further evidence that when patient rights are upheld, patients tend to experience an improvement in their overall well-being. This finding is corroborated by research indicating that respect for patient autonomy and rights has a positive impact on patient satisfaction and quality of care [62–64].

### Implications for practice

The findings of this study underscore the vital necessity for the implementation of augmented training and support programs for nurses in order to equip them with the requisite skills to navigate the ethical challenges that arise in the context of palliative care. Although nurses evince a robust dedication to safeguarding patient rights and ethical standards, they frequently encounter significant obstacles in doing so, particularly in intricate end-of-life scenarios. In order to address these challenges, healthcare organizations should implement a number of targeted strategies. Firstly, the development of comprehensive training programs that focus on ethical decision-making in palliative care is of paramount importance. Such programs might incorporate case-based learning, in which nurses analyze authentic scenarios in order to develop their capacity to navigate ethical dilemmas. Furthermore, periodic workshops and seminars conducted by experts in the field of ethics can facilitate continuous education and foster open discourse on the moral challenges encountered in practice.

Secondly, healthcare facilities should implement mentorship programs that pair less experienced nurses with more senior professionals who have a strong background in ethical issues. Such mentorship can facilitate guidance and cultivate an ethical reflection culture. Thirdly, the integration of ethical consultations into the established framework of palliative care meetings can facilitate a structured approach to addressing ethical concerns. Such an approach would permit nurses to present cases they perceive as challenging and to collaborate with interdisciplinary teams in order to develop solutions that respect the autonomy and rights of the patient.

Ultimately, it is imperative to foster an environment that is conducive to nurses articulating their ethical concerns without apprehension of reprisal. This could entail the implementation of anonymous reporting systems and regular feedback sessions, with the objective of ensuring that nurses feel supported in their ethical decision-making processes. By prioritizing these initiatives, healthcare facilities can enhance the competence of nursing staff and the overall quality of care provided to patients and their families.

## Conclusion

The study emphasises the pivotal role of nurses in negotiating the ethical complexities inherent in palliative care, underscoring the significant correlation between ethical issues, quality of life and adherence to patient rights. The findings demonstrate that nurses encounter substantial ethical challenges that impact their overall well-being and capacity to deliver optimal care. Despite a generally high level of awareness of patient rights, areas for improvement were identified, particularly with regard to patient advocacy and emotional well-being. It is imperative that nurses have access to continuous professional development and support systems in order to empower them to make ethical decisions, which will ultimately enhance the quality of care delivered to patients in palliative settings. It is of the utmost importance to address these factors in order to foster a more compassionate, ethical, and effective palliative care environment.

### Recommendation

It is imperative that healthcare organizations implement comprehensive ethical decision-making frameworks and provide ongoing training for nurses to address the ethical challenges inherent in palliative care. This will ultimately enhance patients’ quality of life. These frameworks should concentrate on equipping nurses with the requisite skills to navigate complex ethical dilemmas, particularly in end-of-life care, while ensuring the consistent protection of patient rights, including autonomy, informed consent, and confidentiality.

Furthermore, healthcare institutions should invest in comprehensive symptom management strategies and interdisciplinary support in order to enhance patients’ physical and social well-being. Specific examples of comprehensive symptom management strategies may include the implementation of pain assessment protocols utilizing validated tools, such as the Numerical Rating Scale (NRS) or the Visual Analogue Scale (VAS), for the regular evaluation and effective addressing of pain levels. Furthermore, institutions could establish specialized pain management teams comprising pain specialists, pharmacists and nurses trained in palliative care, with the objective of developing individualized pain management plans.

Furthermore, the integration of non-pharmacological approaches, such as mindfulness, cognitive-behavioral therapy, and art or music therapy, can address psychological symptoms like anxiety and depression while also promoting overall well-being. The implementation of regular training workshops on these complementary therapies could facilitate the incorporation of these techniques into nursing practice.

In order to facilitate interdisciplinary assistance, organizations may consider the formation of care coordination teams comprising nurses, social workers, chaplains and physiotherapists. The objective of such teams would be to develop holistic care plans that address patients’ physical, emotional and spiritual needs. The convening of regular team meetings would facilitate effective communication and collaborative working across all disciplines, thereby ensuring the provision of optimal support to patients and their families.

In order to maintain patient-centred care and uphold ethical principles in palliative care delivery, regular ethical consultations and case discussions should be integrated into practice. This will facilitate collaborative discussions among nurses and other healthcare professionals regarding challenging cases and the sharing of best practices. The implementation of these recommendations by healthcare organisations will facilitate the creation of a supportive environment that prioritizes ethical decision-making and comprehensive care, thereby improving the quality of life for patients in palliative care.

### Study limitations

It is essential to consider the numerous constraints of this study when assessing the findings. Firstly, it should be noted that the results may be less applicable to larger populations receiving palliative care, given that the sample size was very small and restricted to certain hospital settings. Moreover, the cross-sectional design, which records a single moment in time, makes it more challenging to assess how ethical issues, patient rights, and quality of life have evolved over time or to ascertain a causal relationship between them. Additionally, the self-reported data provided by the participants may be subject to response bias, with nurses potentially overestimating or underreporting specific ethical difficulties or challenges. Finally, the study primarily focused on the perspectives of nurses, excluding those of patients or other medical professionals. This limited the scope of the study and prevented a more comprehensive understanding of the ethical dynamics in palliative care.

## Data Availability

No datasets were generated or analysed during the current study.
